# Development of sandwich ELISA and lateral flow assay for the detection of *Bungarus multicinctus* venom

**DOI:** 10.1371/journal.pntd.0011165

**Published:** 2023-03-30

**Authors:** Ji-Fei Nong, Zhou Huang, Zheng-Zhuang Huang, Jie Yang, Jin-Cheng Li, Feng Yang, Dong-Ling Huang, Fan Wang, Wei Wang

**Affiliations:** Department of Emergency, the First Affiliated Hospital, Guangxi Medical University, Nanning, China; University of Peradeniya Faculty of Medicine, SRI LANKA

## Abstract

Snakebite envenoming adversely affects human health and life worldwide. Presently, no suitable diagnostic tools for snakebite envenoming are available in China. Therefore, we sought to develop reliable diagnostic tests for snakebite management. We conducted affinity purification experiments to prepare species-specific antivenom antibody (SSAb). In brief, affinity chromatography with an antibody purification column (Protein A) was conducted to purify immunoglobulin G from *Bungarus multicinctus* (BM) venom hyperimmunized rabbit serum. The cross-reactive antibodies were removed from commercial BM antivenin by immune adsorption on the affinity chromatography columns of the other three venoms, *Bungarus Fasciatus* (FS), *Naja atra* (NA), and *O*. *hannah* (OH), generating SSAb. The results of western blot analysis and enzyme-linked immunosorbent assay (ELISA) showed the high specificity of the prepared SSAb. The obtained antibodies were then applied to ELISA and lateral flow assay (LFA) to detect BM venom. The resulting ELISA and LFA could specifically and rapidly detect BM venom in various samples with the limits of quantification as 0.1 and 1 ng/ml, respectively. This method could effectively detect snake venom in experimentally envenomed rats (simulating human envenomation), which could distinguish positive and negative samples within 10–15 min. This method also showed promise in serving as a highly useful tool for a rapid clinical distinguishing of BM bites and rational use of antivenom in emergency centers. The study also revealed cross-reactivity between BM and heterogenous venoms, suggesting that they shared common epitopes, which is of great significance for developing detection methods for venoms of the snakes belonging to the same family.

## Author summary

The abundance of snake species and snakebites is a prominent issue in many tropical and subtropical developing countries. Thus, rapid diagnosis and selection of appropriate and effective antivenoms are the concomitant challenges that must be addressed. Neurotoxic snakebites, such as BM bites, usually occur late at night or accidentally, and local symptoms are not obvious after the bite, which results in difficulty in identifying the biting species. Administering a specific antivenom remains the most effective treatment for snakebite envenoming, thereby necessitating reliable, sensitive, and rapid diagnostic kits for BM bites. We constructed a sandwich enzyme-linked immunosorbent assay (ELISA) and a lateral flow assay (LFA) using two antibodies from different species to increase the sensitivity for venom detection and specifically identified BM venom. We found the device to be highly effective in samples of experimentally envenomed rats. The application of LFA will be conducive to appropriate antivenom administration and prevent adverse reactions.

## Introduction

Venomous snakebite remains a prominent public health-threatening problem worldwide, especially affecting tropical and subtropical developing countries and regions [1]. Snakebite envenomation is still a neglected social issue, with a dearth of knowledge about the identification, delayed diagnosis, and appropriate antivenom [2]. The World Health Organization reported that nearly 3 million cases of venomous snakebites occur worldwide annually, resulting in an estimated 81,000–138,000 fatalities [3,4] and about 400,000 disabilities [1,5]. In China, about 210 snake species have been reported, including 67 species of venomous snakes [6,7]. A snakebite epidemiology survey data from the Guangxi Autonomous Region, an epicenter for snakebite in mainland China, carried out by Guangxi Medical University, showed that the lethality rate for BM bites was about 5%, ranking as second only to that of *Ophiophagus hannah* bites in the past decade [8]. BM represents the most virulent snake inhabiting in mainland China owing to its high mortality rate [9]. The other three common *Elapidae* snake species, FS, NA, and OH, have overlapping activity zones in southern China and neighboring countries [10].

BM has a nocturnal habit, many patients are bitten during sleep or are accidentally bitten, which is easily neglected [11,12]. Compared with OH and NA, which are commonly believed neurotoxic (and cytotoxic) *elapid* species in the region, BM snakebites require prolonged ventilator support [13,14]. Respiratory failure developed within 1.5–6.5 h post-bite [15]. A study reported that some venomous snakebites are “dry bites” that do not result in systemic envenomation [16]. Prior to this time point, the lack of obvious symptoms in patients causes difficulty in differentiating the venomous snakebites from dry bites, leading to the delay of antivenom injection and missing of the optimum rescue time. Therefore, the rapid detection kit is of great significance for early diagnosis and administration of antivenom for the highly occult BM snakebites.

The specific monovalent antivenom therapy remains the preferred treatment for snake envenomation [17]. The administration of monovalent antivenom against snake-specific species could help achieve the fastest recovery and fewer side effects [18]. However, clearly identification of the species in some snakebite incidents is difficult, and currently, the lack of rapid and reliable diagnostic methods makes it impossible to provide specific antivenom treatments [19]. Besides, non-venomous snakebites are a common occurrence [13]. Patients receiving injections of non-specific antivenoms will have an increased risk of adverse reactions [20], and an overdose of antivenom can cause severe allergic reactions [21]. Altogether, the lack of rapid, reliable diagnostic kit has limited the use of antivenom, and delayed treatment results in severe disability and even death among patients [22].

Detecting snake venom proteins using antibodies is a simple and effective method, making it possible to identify the biting species [23]. So far, multiple methods have been developed for detection, including ELISA, immunofluorescence assay, immunoelectrophoresis, agglutination assay, radioimmunoassay, and immunodiffusion, among which ELISA appears to be the most practical and can be easily modified to kits and adapted for use [24–26]. In the assays, crude or partially purified antibodies may lead to diagnostic ambiguity [27]. The main cause of ambiguity was that different venoms may share common antigenic epitope, and antigens are present in the venoms of related and even unrelated snake species [28,29]. To improve the specificity of venom detection, it is necessary to screen antibodies with higher specificity. Initially, it was proposed that monoclonal antibodies against a single toxin in the venom could avoid cross-reactivity [24,30]. Immunology-based biosensors were also developed to detect β-bungarotoxin, a single toxin, by Dong et al. [31]. However, the venom yield of BM is only 4.6 mg on average [7,32], which is lower than other venomous snakes, making venom assay in serum samples more challenging. Pharmacokinetics studies have shown that most single toxins are rapidly eliminated from blood circulation, which restricts the use in the detection [33]. These pose a challenge for the specificity of detected methods. A number of previous studies showed that cross-reactivity antibodies were removed from antiserum through affinity column coupling heterologous snake venoms by affinity chromatography, then the obtained SSAbs were used for the ELISA [34–37]. However, ELISA procedures are time-consuming and require specialized equipment. Compared with ELISA, the lateral flow assay (LFA) involves fewer processing steps, and the results are available within 5–20 minutes. Furthermore, the latter does not require any auxiliary equipment [38]. Therefore, ELISA and LFA have been recognized as the ideal strategies for the identification of snakebites [23,39,40]. Collectively, developing an LFA for rapid detection of BM venom were the chief purposes of this study.

In this study, we produced high-affinity capture antibodies and SSAbs targeting BM venom proteins and then constructed an LFA kit by conjugation with gold nanoparticles based on immunochromatography. The kit with sensitive detection limit, hopefully, practical and suitable for use by clinicians.

## Material and methods

### 2.1 Ethics statement

All the experiments on animals were performed in accordance with the ethical guidelines of the Institutional Animal Ethics Committee and has been reviewed and approved by the Medical Ethics Committee of the First Affiliated Hospital of Guangxi Medical University (Approval ID: 2022-KY-E-(230)).

### 2.2 Materials

Lyophilized venoms of BM, FS, NA, and OH were provided by Institute of Snake Venom, Guangxi Medical University and preserved at −20°C before use. The commercial monospecific BM antivenin (batch no. 20210101, expiry date: 04/01, 2024) was produced against BM and then digested by gastric enzymes provided in equine immune globulin F(ab′)2, which were purchased from Shanghai Serum Biological Technology Co., Ltd. Freund’s complete adjuvant (FCA), Freund’s incomplete adjuvant (FIA), goat anti rabbit immunoglobulin G (IgG)–horseradish peroxidase (HRP) conjugate, HRP-conjugated AffiniPure goat anti-horse IgG (H+L), and bovine serum albumin (BSA) were purchased from Solarbio, China. Tetramethyl benzidine–hydrogen peroxide (TMB/H_2_O_2_) substrate and prestained color protein marker were purchased from Beyotime, China. The Protein A Magbeads (L00273) and High-Affinity Antibody Purification Kit (L00404) were purchased from GenScript, China. The HRP Quick Labeling Kit was purchased from Frdbio, China. A nitrocellulose (NC) membrane of pore size 0.44 μm was obtained from Immobilon, USA. Adult male rats (200–250 g), male Swiss albino mice (18–22 g), and New Zealand white male rabbits (2.0–2.5 kg) were maintained in a controlled environment with a dark/light period of 12 h at a temperature of 22°C and a humidity level of 60%–70%. All other chemicals and reagents used were of analytical grade.

### 2.3 Estimation of protein content

The protein concentration of antibodies and venom were determined via Bradford’s method [41]. Equine immunoglobulin and BSA were used as standard for antibodies and snake venom estimations, respectively.

### 2.4 Detoxification of venoms and immunization

#### 2.4.1 Detoxification of venoms and determination of lethal dose 50 (LD_50_)

Rabbits were immunized with detoxified venom of BM according to the method described by Shaikh [42] with slight modifications. The BM venom was diluted in sterile normal saline (1 mg/ml), heated for one hour at 55°C, and immediately chilled in ice/water mixture for 10 min. This process was repeated twice. The detoxified venom LD_50_ was performed in accordance with the WHO’s guidelines for the production, control, and regulation of snake antivenom immunoglobulin (2010). Briefly, a group of six mice (18–22 g) were injected intravenously with 0.5 ml of graded detoxified venom dilutions (1.0 μg/ml to 2.5 μg/ml) by assuming the nominal LD_50_ of detoxified venom in sterile physiological saline. Survival and deaths of 24 h were recorded, and the LD_50_ values of the detoxified venom were estimated according to the method described by Reed and Muench [43,44].

#### 2.4.2 Immunization

Before immunization, 5 ml of pre-immune rabbit serum was gathered from normal rabbits subsequently preserved at −80°C. Two New Zealand white male rabbits were inoculated separately with a sub-lethal dose of BM venom to produce venom-specific monovalent antiserum. Each rabbit was injected with 1.0 ml of dose subcutaneously on the back at a specific time interval. First, detoxified BM venom (100 μg/kg body mass) emulsified with FCA was given subsequently two dosages with IFA as an adjuvant injected every 15 days. Further, the fourth and fifth booster dosages of crude venom (50 and 100 μg/kg body mass) mixed with 2% sterile bentonite were administered. The last two progressive dosages of BM venom (100 μg/kg body mass) prepared in sterile physiological saline were inoculated. The antibody titer in rabbit plasma was monitored with indirect ELISA 10–12 days after each dose.

### 2.5 ELISA for the determination of rabbit antibody

Indirect ELISA was performed per the method described by Lee [18]. Briefly, BM venom was diluted to 2 μg/ml with a coating buffer (0.1 M sodium carbonate/bicarbonate, pH 9.6) and then loaded onto the 96-well polystyrene microtiter plate, 100 μL per well, and finally incubated overnight at 4°C. After washing thrice with 350 μl phosphate buffer saline containing 0.05% Tween 20, pH-7.4 (PBST), the wells were blocked with 250 μl of the blocking solution (2% BSA in PBST) for 1 h at 37°C. After three rinses with PBST, 100 μl of gradient-diluted immunized rabbit serum in sample buffer (1% BSA in PBST) was added into each well and incubated for 1 h at room temperature on an orbital shaker. Afterward, each well was thoroughly washed with the PBST buffer and then incubated in 100 μL of HRP-conjugate goat anti-rabbit secondary antibody (1:10000 diluted in PBST) for 30 min at room temperature. The unbound secondary antibody was removed after washing five times and then 100 μl of TMB/H_2_O_2_ was added and incubated for 15 min and kept from light. Next, each well was added with 50 μL of 2 M H_2_SO_4_ to terminate the reaction, and the absorbance was measured immediately at 450 nm via an ELISA plate reader (BioTek ELx 808, USA). All the values indicated in results are blank corrected.

### 2.6 IgG purification

Rabbit hyperimmune plasma raised against BM with antibody titer higher than 100,000 as detected via indirect ELISA, were subjected to antibody purification. The blood samples were collected by heart puncture of each rabbit separately in tubes containing 5% sodium citrate and then centrifuged at 5000 g for 5 min to obtain clear plasma. Furthermore, as per the instruction manual of the Protein A kit (L00273 GenScript), the affinity-purified antibody (APAb) was obtained by immunoaffinity adsorption and purification of the IgG fraction and then preserved at 4°C.

### 2.7 SSAb purification

**Fig 1:**
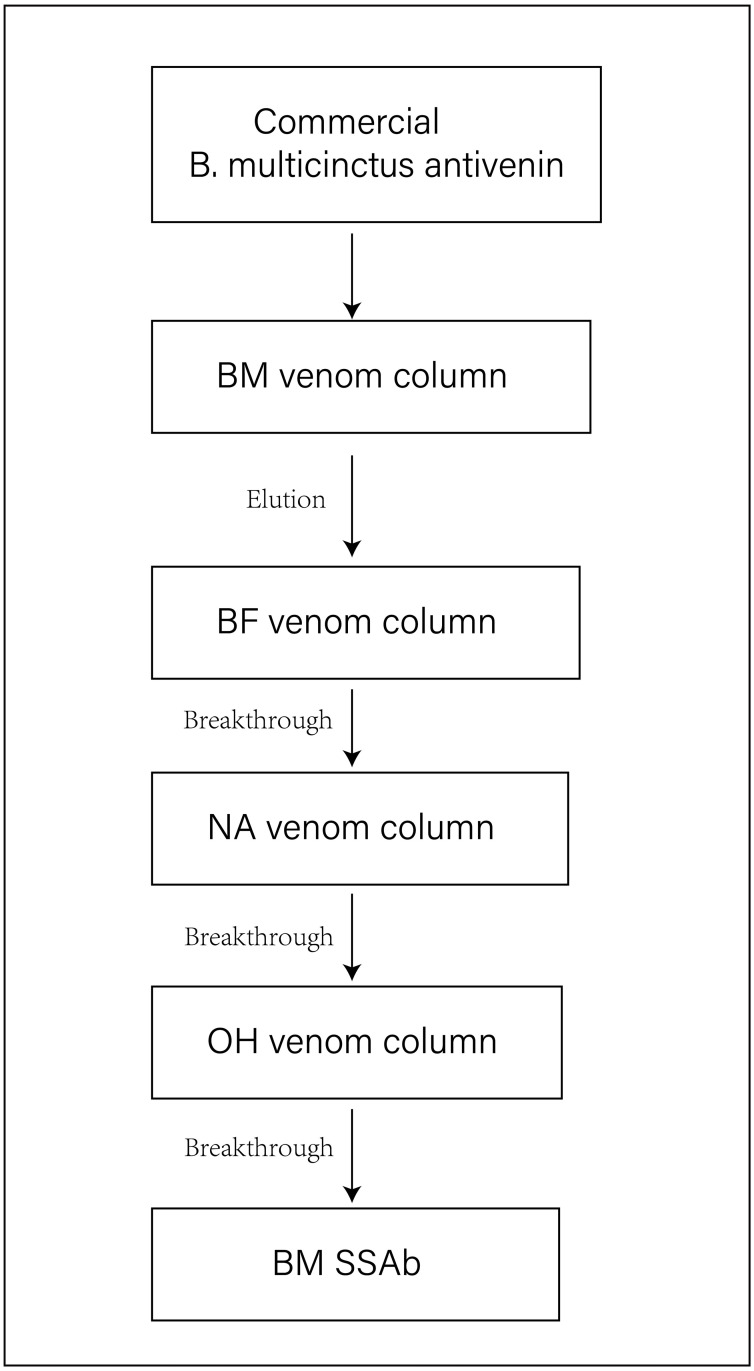
SSAb Preparation. Two-step immuno-affinity purification of BM SSAb: step 1, the commercial BM antivenin was absorbed by BM venom column and then eluted. Step 2, SSAb was obtained by immuno-affinity chromatography with other three heterologous venom columns to remove cross-reactivity antibody components.

The conspecific SSAb against BM were prepared using the method introduced by Lee [37], with slight modifications. Commercial BM antivenin was subjected to immuno-affinity chromatography with four venom columns, which were respectively coupled with four venoms onto the pre-activated High-affinity Iodoacetyl Resin (L00404, GenScript, China) by following the manufacturer’s instructions. In brief, the pre-activated resins were packed into the columns and incubated separately with the four snake venoms dissolved in binding buffer (50 mM tris-HCl, 5 mM EDTA-Na, pH 8.5) overnight at 4°C on a shaker for coupling of venom components onto resins. After washing with the binding buffer, the active sites on the resins were blocked with the blocking buffer (50 mM L-Cysteine•HCl in the coupling buffer) overnight at 4°C on a shaker. Afterward, the resins were washed with the washing buffer (PBS, pH 8.0). Next, BM antivenin was applied on the first affinity column, which contained BM venom, at 4°C for 6 h on a shaker. The unbound antibody was rinsed thoroughly with PBS, and then bound antibody was rinsed with the elution buffer (100 mM glycine•HCl, pH 2.0–2.5). After that, the flow-through solution was equilibrated with the neutralizing buffer (1 M Tris•HCl, pH 8.5) and then sequentially passed through the other three venom affinity columns at 4°C for 6 h. The flow-through solution was then dialyzed against PBS and stored at −20°C. The experimental procedure schematic for purification is presented in Fig 1.

### 2.8 SSAb Conjugation

The BM SSAb was labeled with an enzyme HRP based on the manufacturer’s protocol (Frdbio, China). Briefly, the SSAb solution (2.0 mg/ml) was mixed well with 1/10th volume of the labeling buffer, transferred into the tube containing the same amount of HRP powder, and then mixed well, followed by 2 h of incubation at room temperature and protected from light. Subsequently, the reaction was halted by mixing well with 1/10th volume of the reaction termination buffer at room temperature for 1 h. The enzyme-labeled antibody was stabilized immediately with the stabilizer solution of the same volume and stored at −20°C.

### 2.9 Immunoblot analysis

The four crude venoms and antibodies were first runed on SDS/12.5% (w/v) polyacrylamide gel under reducing condition as per the method presented by Laemmli [45] and then either stained with Coomassie blue R-250 or transferred onto a PVDF membrane (0.22 μm, Millipore). The membrane was then blocked with 5% nonfat milk in the washing buffer (TBST, 20 mM tris buffered saline pH 7.6 containing 0.05% Tween-20) at room temperature for 1 h on a horizontal shaker. After washing with TBST, the PVDF membrane was incubated with suitably diluted commercial antivenin/SSAb (1mg/ml) in TBST overnight at 4°C on a horizontal shaker. Next, the PVDF membrane was subsequently rinsed in TBST to remove the primary antibody and then incubated with HRP-labeled anti-horse IgG 1 h at room temperature. After the unbound secondary antibodies were thoroughly washed off, the membrane was immersed in peroxidase chromogenic substrate solution and then visualized immediately using the FluouChem HD2 (ALPHA, USA).

### 2.10 Experimental envenomation study

Thirty rats within a defined weight range (200–250 g), divided into five groups equally, were injected subcutaneously with 0.414 μg/g body mass of BM venom dissolved in 200 μl of normal saline. Furthermore, six normal rats injected with an equal volume of normal saline served as negative controls. After oral endotracheal intubation, animals of the experimental group were mechanically ventilated until the observation time point. All experiments on animals were performed in accordance with the instructions by Wolfensohn and Lloyd [46], and all efforts including comfortable circumstance, appropriate anesthesia and experience were made to minimize their suffering. The animals were injected intraperitoneally with 3% sodium pentobarbital for satisfied anesthesia. Blood samples were collected by abdominal aorta puncture into serum tubes, and tissues (liver, brain, and muscle from the injection site) were collected for the assay of venom at various time intervals: 0, 1, 2, 4, 6, and 8 h (n = 6). Afterward, blood samples were centrifuged at 3000 × g for 15 min at room temperature, and then, the supernatant was stored at −20°C until use. When the rats urinated, urine samples were collected and preserved at −20°C until being tested. The blood contamination of tissues was eliminated by washing with PBS and then redundant buffer was removed with filter paper. Subsequently, tissues were weighed, added with equal amount of PBS, and then homogenized with a cryogenic grinder (Servicebio KZ-III-F, China) for 2 min. Finally, tissue homogenates were centrifuged at 12,000 rpm for 15 min at 4°C. The clear supernatant was then analyzed by ELISA and LFA.

### 2.11 Sandwich ELISA assays for the determination of venoms

The rabbit APAb solution (2 μg/ml) was prepared with the coating buffer (0.1 M sodium carbonate/bicarbonate, pH 9.6) and loaded 100 μl each well onto polystyrene micro-ELISA plates, then incubated at 4°C for overnight and rinsed thrice with 350μl PBST. Furthermore, the wells were sealed with 250 μl of the blocking buffer (PBST containing 2% BSA) for 1 h at 37°C. After rinsing thrice with PBST, 100 μl tissue samples, known concentrations of four venoms (0.01 to 1000 ng/ml) in the sample buffer (1% BSA in PBST), were added into the plate and incubated for 1 h at room temperature on an orbital shaker. Afterward, each well was washed with PBST, and 100 μl solution of the HRP-labeled SSAb (1 mg/ml, equine origin) 1:10,000 diluted in PBST were added and incubated for 30 min at room temperature. After washing five times, 100 μl of TMB/H_2_O_2_ was added to each well and incubated in the dark at room temperature for 15 min. Then, each well was added with 50 μL of 2 M H_2_SO_4_ to terminate the reaction, and the absorbance was measured at 450 nm with the ELISA plate reader (BioTek ELx 808, USA). All the values indicated in results are blank corrected. Control wells were coated with BSA as a negative control. All measurements tests were done in triplicate. Titration curves were constructed by plotting logs of venom concentration against absorbance to quantify the venom content of the test samples. Quantitative data are expressed herein as the means and standard deviations (SD) of the readings obtained from control specimens.

### 2.12 Cross-reactivity evaluation of the commercial antivenin

The cross-reactivity of the commercial BM antivenin was assessed via indirect ELISA. The ELISA plates pre-coated with four snake venoms were individually reacted with serially diluted commercial antivenin (1,000–128,000). The steps involved in this process are the same as those described above in section 2.5, but the HRP-conjugated AffiniPure goat anti-horse IgG (H+L) was used as the secondary antibody.

### 2.13 Development of lateral flow strips

#### 2.13.1 Synthesis of colloidal gold

Gold nanoparticles (40 nm) were prepared using a previously proposed method [47], according to which 50ml HAuC1_4_ (0.01%) solution was heated to boiling, and 500 μl sodium citrate (1%) was rapidly added while vigorously stirring for 15 min. The colloidal solution color changed to gray, subsequently turned black, and gradually stabilized to red. The colloidal solution was chilled to room temperature and preserved in clean brown bottles at 4°C until use. The absorption peak of the solution was monitored at 533 nm using an ultraviolet–visible spectrophotometer (UV-5500 [PC] Metash China).

#### 2.13.2 Preparation of colloidal gold probes

The rabbit APAb was adjusted to 1.0 mg/ml, while the gold solution pH was adjusted to 7.0–8.0 with 50 mM potassium carbonate buffer (pH 9.6), and then, the rabbit APAb was added to the gold solution and mixed well at 37°C for 15 min. To block the unbound sites, BSA was added up to 1% and centrifuged at 13,000 rpm for 30 min via hypothermic high-speed centrifuge (Eppendorf, Germany). Afterward, the colloidal gold-conjugated antibody was resuspended in the storage solution (PBS containing 1% BSA, 0.01% PEG6000 and 5% sucrose) and stored at 4°C.

#### 2.13.3 Development of LFA strip

The LFA strip included four major parts, namely, sample pad, conjugation pad, NC membrane, and absorption pad, assembled in a specific order. The conjugation pad was saturated with the colloidal gold-conjugated APAb and then dried for 2 h at 37°C. The SSAb (1.0 mg/ml, equine origin) and goat anti-rabbit antibody were dispensed onto the NC membrane as test line and control line, respectively. Each line was 5 mm apart. Membranes were then blocked with blocking buffer (PBS containing 1% BSA) for 30 min, dried for 2 h at 37°C. Finally, the membrane was immobilized on the cardboard, followed by conjugation and absorption pads that overlapped with the membrane on each side about 2 mm. These strips were protected from sunlight and humidity and stored at room temperature.

#### 2.13.4 Venom detection with lateral flow strips

Each venom was diluted in PBS, ranging from 0.01 to 1000 ng/ml, and samples were analyzed by applying 100 μl on LFA with PBS as a blank. Additionally, all samples collected from envenomed rats were subjected to LFA and analyzed by applying 100 μl solution on the strips. Normal samples were set as a blank control. After 10 min, the results were recorded.

### 2.14 Data analysis

Statistical analysis was performed with SPSS statistics 17.0. Descriptive analysis of the results was performed by calculating mean values ± SD. All ELISA results were interpreted using GraphPad Prism 9 software.

## Results

### 3.1 Surveillance of immunization

**Fig 2:**
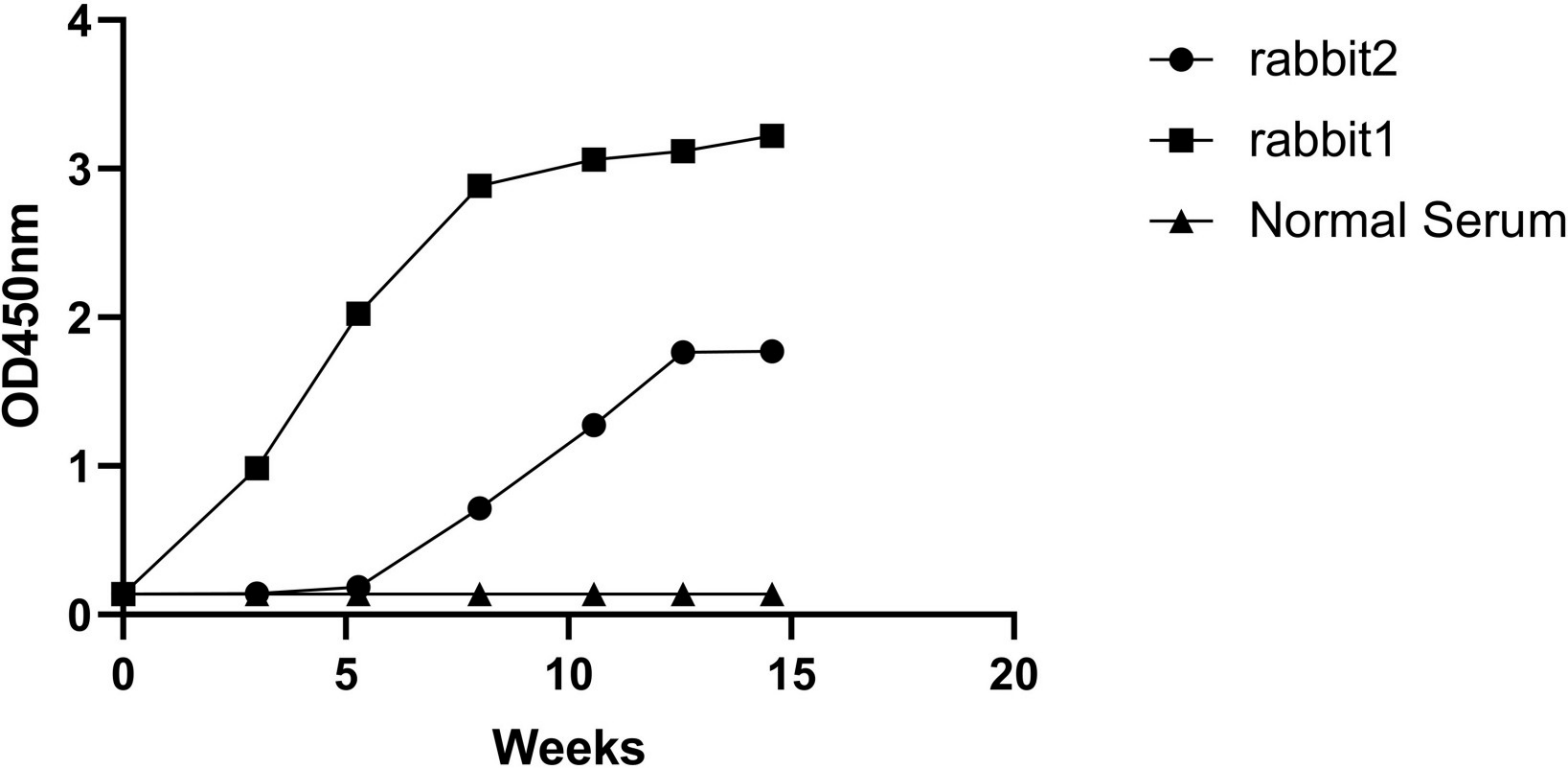
Immuno-conversion of BV, pre-immune rabbit plasma was used as negative control. The OD values (n = 3) are presented as mean ± SD.

Results of animal immunization suggested that the immune response enhanced remarkably after the third and fourth immunization and reached a plateau in comparison with pre-immunization. The obtained antibody titer was stably higher than 100,000 as detected by Indirect ELISA. Initiating immunization using detoxified venom allowed for higher doses of antigen without adverse effects on the animals. The LD_50_ of detoxified venom in mice (s.c.) was 280 ug/g, over 3000 times higher than that of the crude venom, 0.09ug/g. Animals received a higher dose of immunogen, which could significantly raise the titer of antibody and obtained high-affinity capture antibody. Antibodies were generated against crude venom, even though detoxified venom was used for immunization (Fig 2). The relatively higher plasma of rabbit was subjected to IgG purification.

### 3.2 Venom electrophoretic profiling

**Fig 3:**
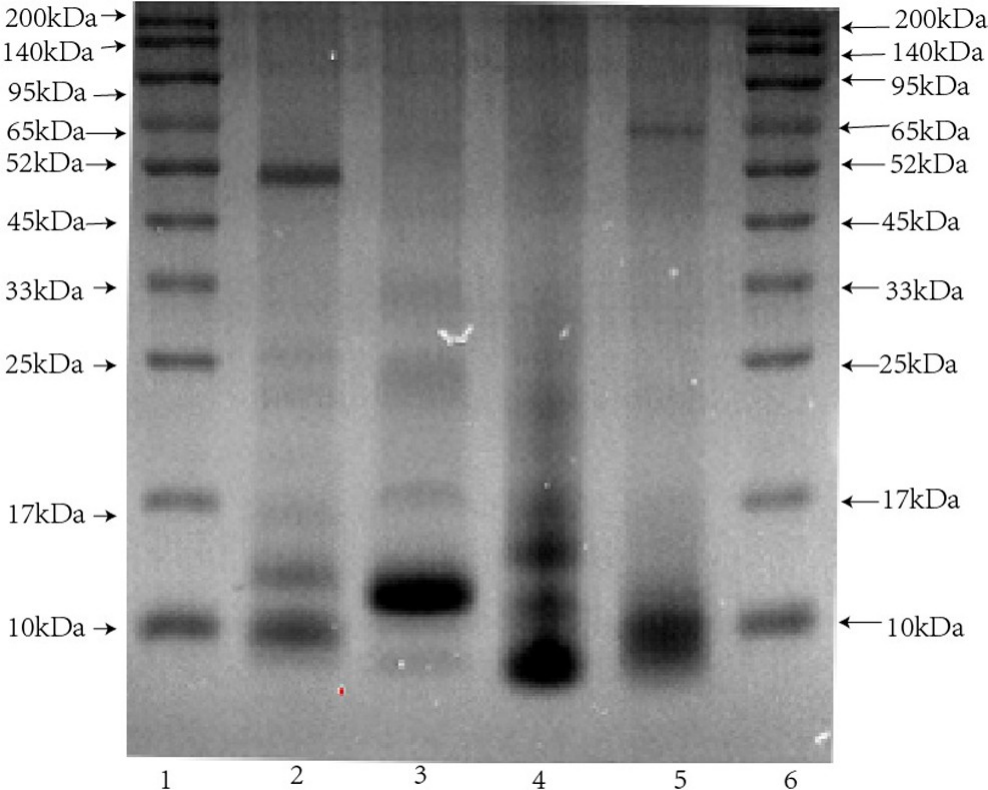
Electrophoretic profile of four venoms. SDS-PAGE (12.5% reducing condition) performed to assess the protein profiles of four venoms, and bands stained with Coomassie brilliant blue G-250. Lanes 1 and 6: standard protein marker, indicating protein MW marker. Lanes 2, 3, 4, and 5 depict BM, BF, NA, and OH venoms, respectively. The arrows depict the MW in kilodalton (kDa).

SDS-PAGE (12.5% reducing condition) was performed to assess the protein profiles of four venoms, and the resulting bands were visualized via Coomassie staining. As evident in Fig 3, venoms had unique but overlapping band patterns such that abundant molecular weights (MWs) of these bands were less than 25 kDa, which indicates the presence of proteins with similar MWs in crude venoms. In addition, comparing the protein abundances, BM and OH showed more distribution in medium-MW range (50–70 kDa) compared with those of FS and NA.

### 3.3 Purity evaluation of APAb and SSAb

**Fig 4:**
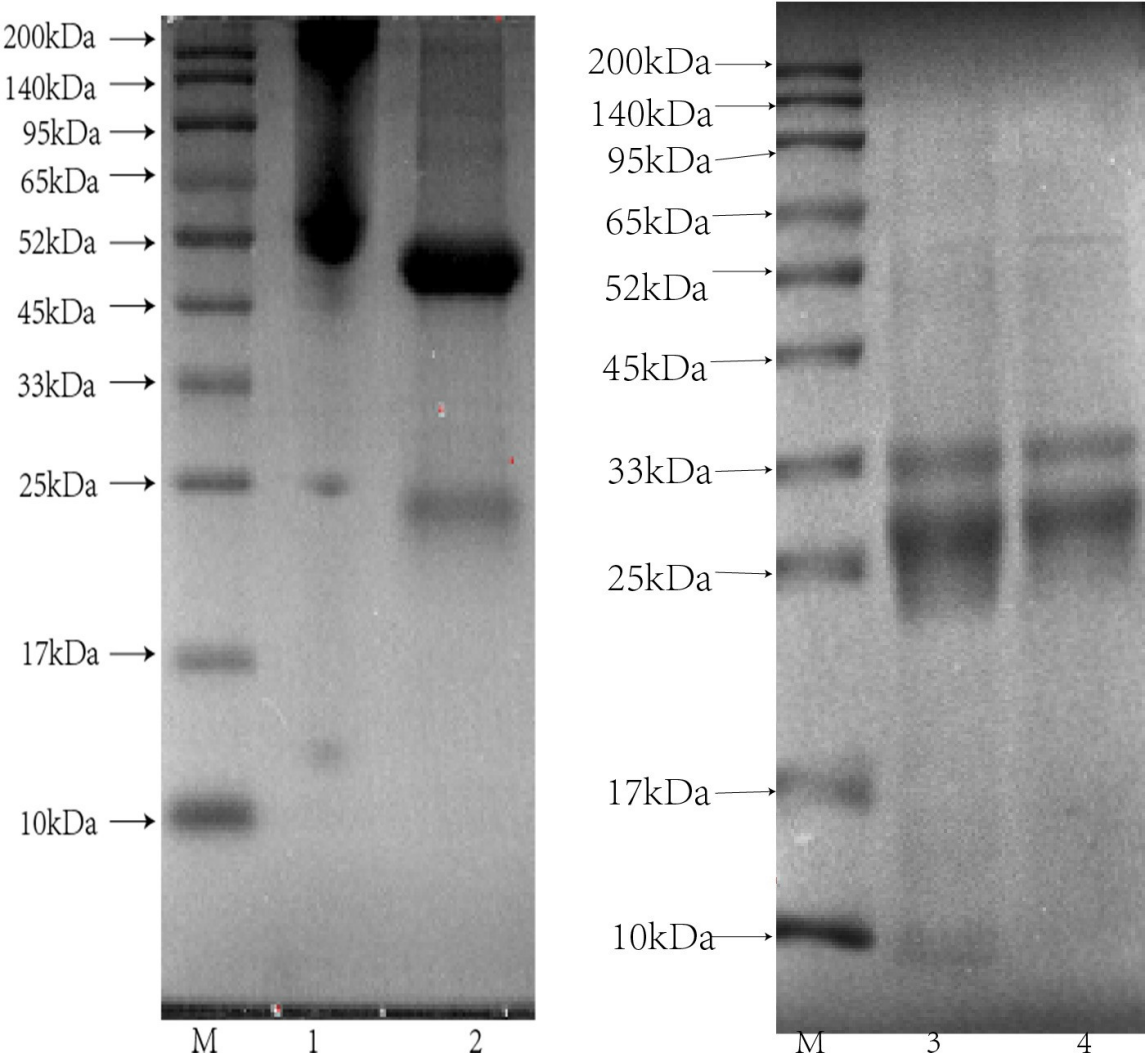
Purity evaluation of affinity purified antibodies. SDS-PAGE (12.5% reducing condition) performed to resolve proteins, and Coomassie staining was used to visualize the resolved bands. M: standard protein marker, lane: 1 and 2 represent rabbit monovalent serum raised against BM venom and its purified antibodies (APAb).

The protein profiles of monovalent rabbit antiserum, APAb, commercial BM antivenin, and SSAb were assessed on SDS-PAGE (12.5% reducing condition). Next, the resulting bands were stained and visualized with Coomassie brilliant blue G-250. The results showed that monovalent rabbit antiserum contained miscellaneous bands pattern, whereas the affinity purified antibody contained clear bands, which is a typical pattern of IgG heavy and light chains, thereby confirming the purity of the antibody. The protein profiles of SSAb showing two clear dense bands respectively belonged to IgG heavy and light chains of equine immune globulin F(ab′)2, and the overall MW was approximately 110 kDa (Fig 4).

### 3.4 Cross-reactivity assessment of commercial monovalent antivenin

**Fig 5:**
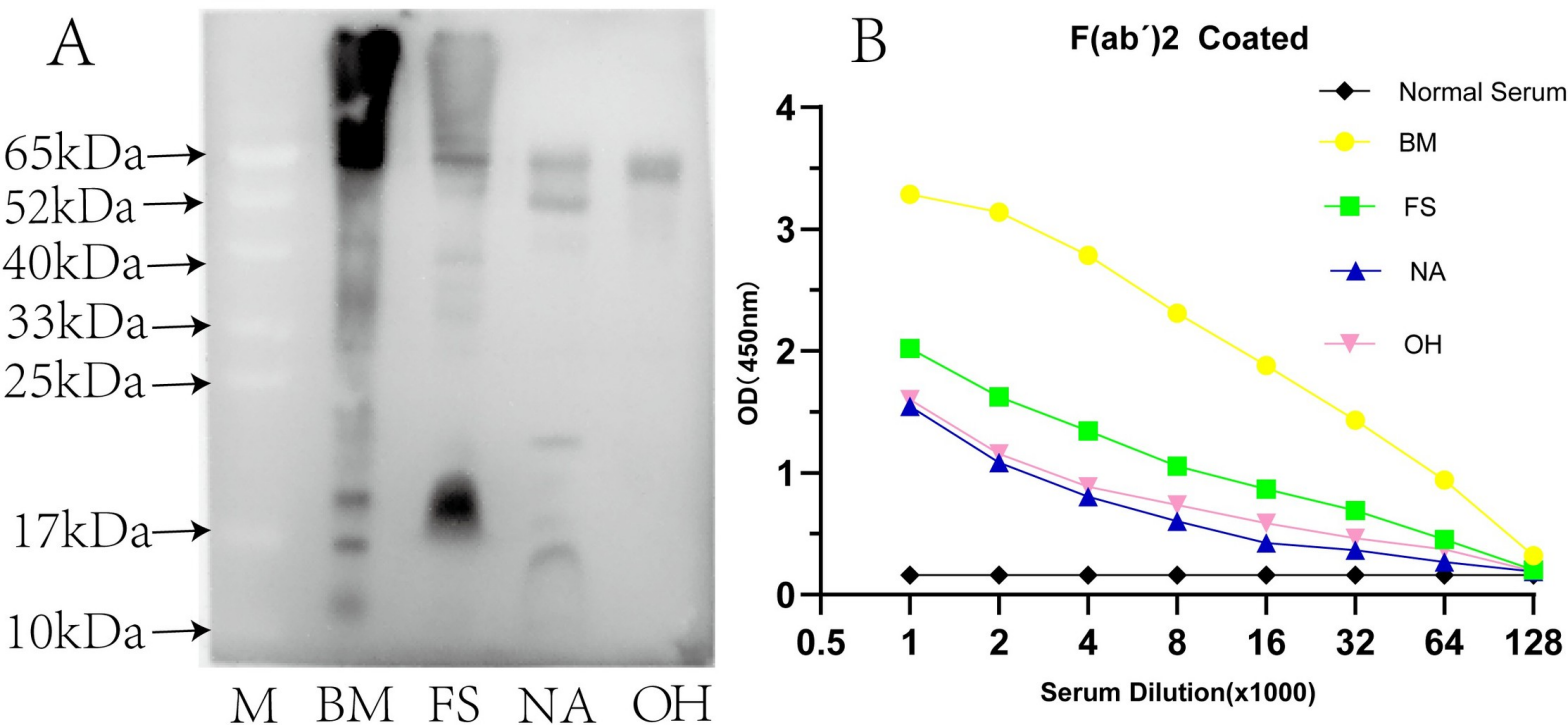
Cross-reaction assessment via western blotting and indirect ELISA. (A) The venoms of BM, FS, NA, and OH were treated with SDS-PAGE sample buffer in reducing conditions and transferred to the PVDF membrane and hybridized by commercial BM antivenin. The arrows represent the MW in kilodalton (kDa). (B) Venom samples were coated on the 96 microplates and detected by commercial BM antivenin in gradient dilutions. All tests were assayed in triplicate and normal horse serum (×) was used as the negative control. All values are presented herein as mean ± SD.

The cross-reactivity of commercial antivenin with four venoms were detected via the western blot and sandwich ELISA assays. The profile of the western blot assay indicated that a number of bands, especially the low-MW bands of FS and NA (10–25 kDa), high-MW bands of FS and OH (52–200 kDa) cross-reacted to commercial antivenin (Fig 5A). The vast majority of BM bands showed high immunogenicity. The reactivity of antivenom toward homologous venom was stronger than that toward heterologous venom. Although the low-MW components are abundant in venoms of the *Elapidae* family (Fig 3), medium and large venom components show more immunogenicity. In SDS-PAGE, some protein bands of venoms (e.g., ~17.65 kDa in FS and ~52.65 kDa in NA) were light or sightless, but they showed significant immunogenicity in immunoblot results.

Positive signals were detectable amongst commercial monovalent antivenin and heterologous venoms in the ELISA test. The ELISA results showed that the OD value of the cross-reaction was gradually raised with the increase in serum antibody concentration (Fig 5B). The cross-reactivity intensities were more prominent between commercial BM antivenin and venom of FS, than the NA and OH. Cross-reactivity analysis also revealed that strong reaction activities occurred amongst venoms from the same family with commercial BM antivenin. The observed cross-reactivity could result in the ambiguous diagnosis during snake species identification.

### 3.5 Specificity of SSAb

**Fig 6:**
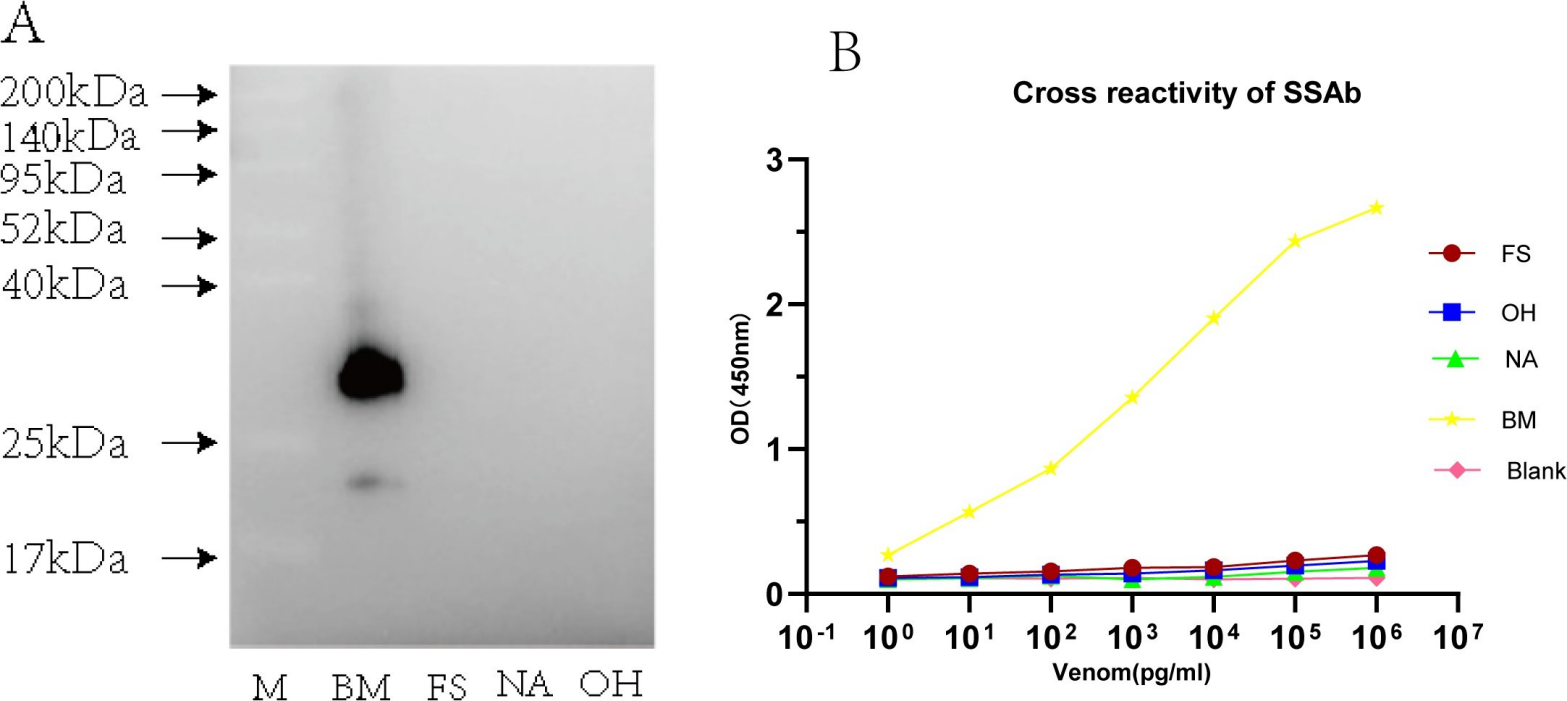
Specificity assessment of SSAb by Western blot and sandwich ELISA. (A) The venoms of BM, FS, NA, and OH were transferred to the PVDF membrane and hybridized by SSAb screened from commercial BM antivenin. The arrows represent the MW in kilodalton (kDa). (B) The rabbit APAb (2 μg/ml) as the capture antibody was incubated with BM venom (range of 0.001–1000 ng/ml). BSA was used as a blank. The OD values (n = 3) are presented herein as mean ± SD.

In this study, cross-reactive antibodies in antivenom were eliminated by immuno-affinity chromatography. The immunological specificity of the SSAb was determined with four venoms at different concentrations by sandwich ELISA, and a suitable concentration of the SSAb was tested with each venom resolved in SDS-PAGE by the western blot assay. A band around 35 kDa, which was immunodetected, should be an important, exclusive antigen for BM venom. Consistent with the results of western blot analysis, the sandwich ELISA results confirmed that SSAb was specifically against the BM venom, and no cross-reaction was observed over a wide concentration range of the other three resolved venoms (Fig 6).

### 3.6 Sensitivity of sandwich ELISA for BM detection

**Fig 7:**
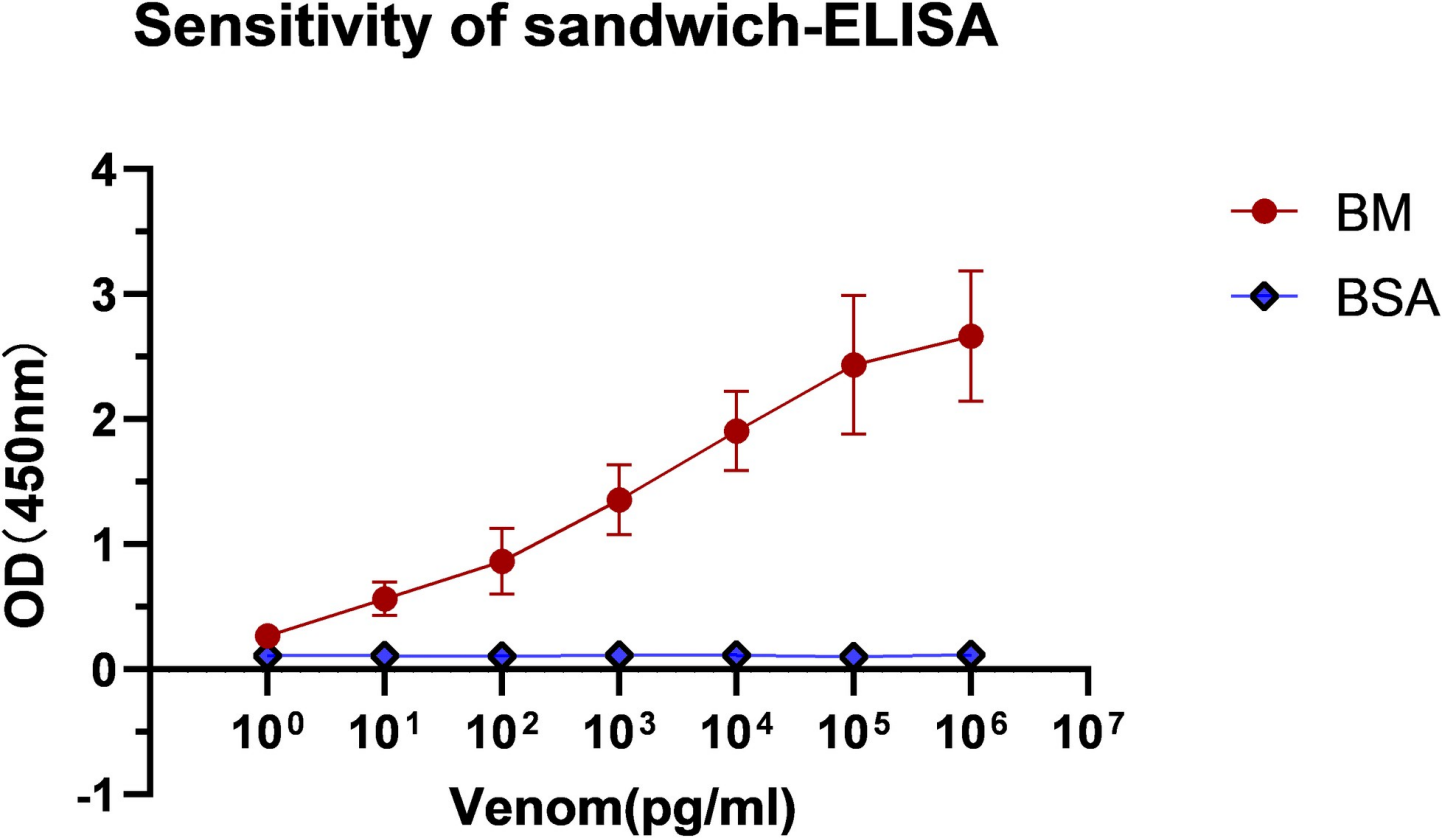
Sensitivity of sandwich ELISA at spiked venom samples. The rabbit APAb (2 μg/ml) as the capture antibody coated on a micro-ELISA plate. Known concentrations of BM venom (0.001–1000 ng/ml) were applied and then tested with SSAb (1 mg/ml) 1:10,000 diluted in PBST. The test was done in triplicate, and BSA was used as a blank. Quantitative data are presented herein as mean ± SD.

Logarithmic dilution of BM resolved in PBS (ranging from 0.001–1000 ng/ml) was detected via sandwich ELISA to determine the sensitivity. The assay was performed in triplicate using BSA as the negative control. ELISA cutoff value was defined by the mean plus three standard deviations of the OD obtained from the control sample, and the minimum detection limit was 0.1 ng/ml (Fig 7).

### 3.7 Venom detection in envenomed rats

**Fig 8:**
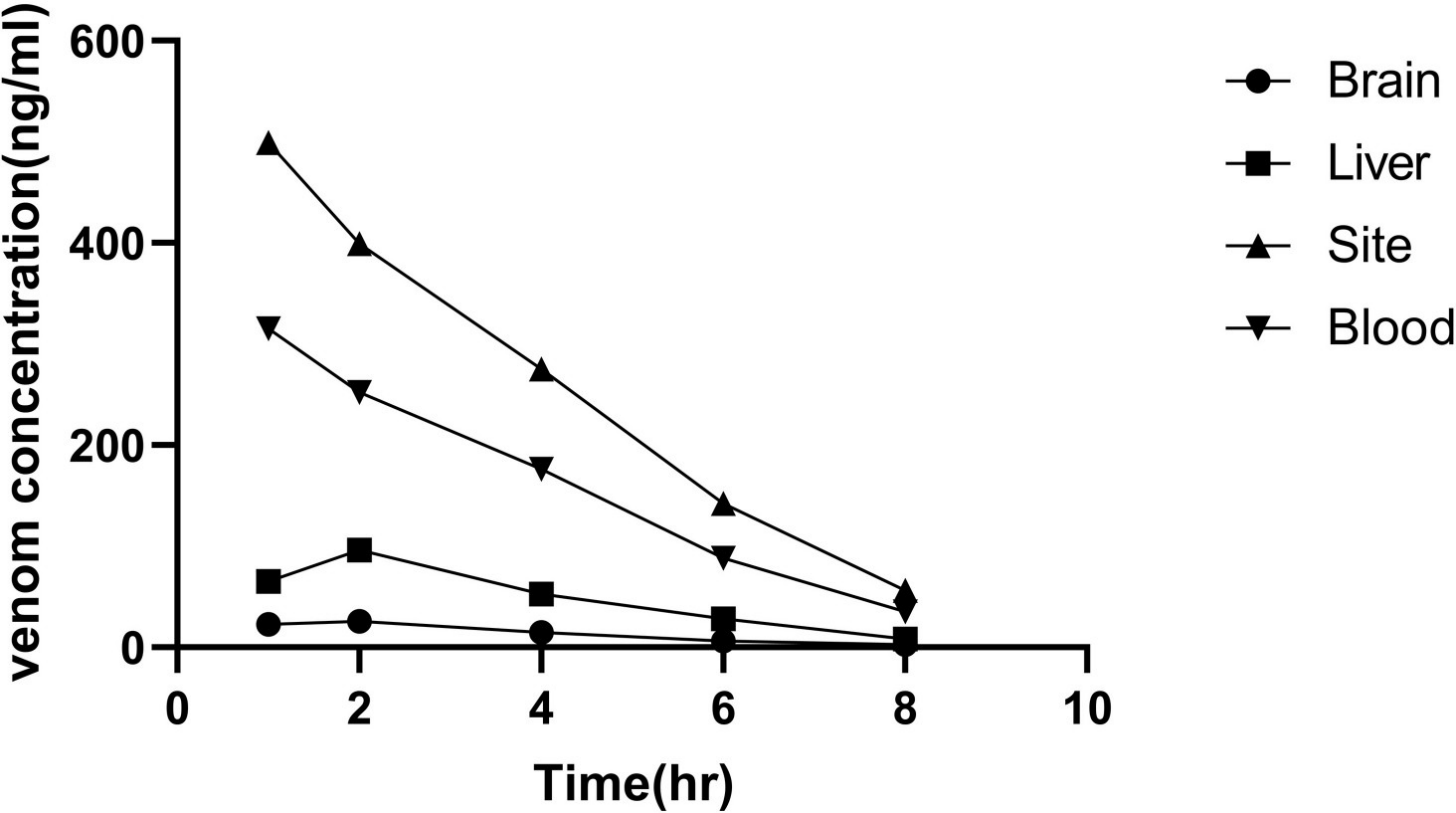
Kinetics of the BM venom injected in rats (simulated amount of snake venom at a snakebite dose in human): Crude BM venom in normal saline (200 μl) was injected subcutaneously to the back leg of the rats. The supernatants of tissue samples and serum were collected at five time points (1, 2, 4, 6, and 8 h) post injection. Data are presented herein as mean ± SD from six rats in each group. All samples were analyzed in triplicate.

All rats presented local symptoms within 5–10 min after the injection of BM venom. In the beginning, the leg on the injected side developed paralysis, and after 1 h, rats developed systemic neurotoxic symptoms and severe neuromuscular paralysis, and exudation from the airway was observed, after which breathing became difficult. Ventilatory support was available via endotracheal intubation, and all rats survived until all time points for observation. Postmortem examination showed no evidence of subcutaneous hemorrhage around the injected area and peritoneal hemorrhage. Kinetics of the venom in blood showed that the rapid distribution of toxins reached a peak at 1 h post injection corresponding to the time when animals presented symptoms of neurotoxic envenoming, and venom was detectable in all blood samples. The highest concentration was that of the supernatant of the injected site homogenate detected post 1 h after the injection (Fig 8). The venom could also be detected in tissue and urine samples collected at individual time points. Samples taken from the control group showed a negative signal. Altogether, the obtained results demonstrated that the developed sandwich ELISA can specifically identify and quantify BM venom in vivo.

### 3.8 Assay performance of LFA strip

**Fig 9:**
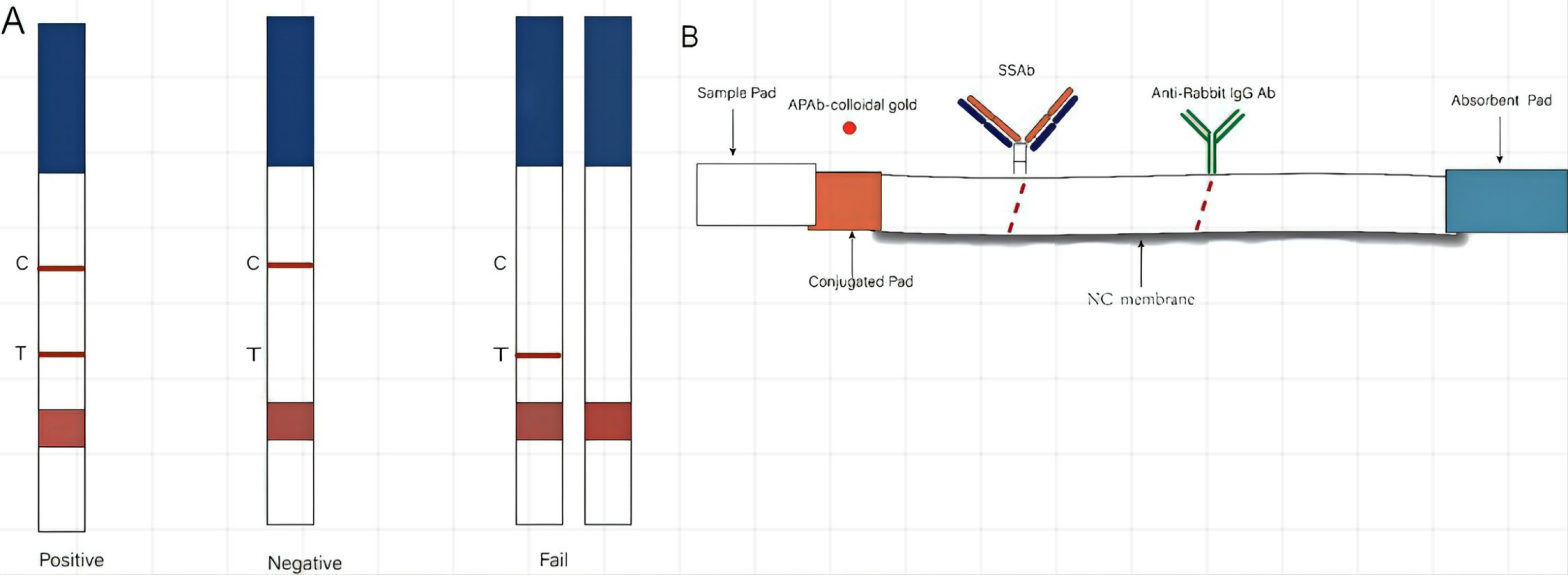
Schematic diagram of the LFA for detecting snake venom. (A) A schematic illustration of the predicted results is displayed. C, control line; T, test line; and (B) The design of the LFA strip constructed on the basis of IgG-conjugated colloidal gold and SSAb.

**Fig 10:**
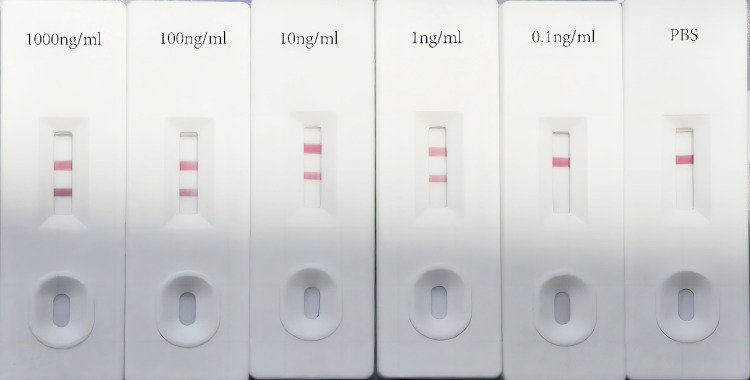
BM venom serially diluted to 1000 to 0.1 ng/ml in PBS and then applied to the LFA for determination of detected ability. Left to right: serial dilutions of applied venoms (1000 to 0.1 ng/ml). PBS used as the negative control. The sensitivity of device was up to 1 ng/ml, as the red line can be seen visually on the strip number 4 in the test region.

Though sandwich ELISA presented high sensitivity and precise quantification, its performance was limited by the multiple steps and long duration. LFA was developed to optimize the procedure of snakebite identification. The colloidal gold-labeled APAb was pasted to the cardboard, while SSAb and goat anti-rabbit IgG antibody were immobilized on the NC membrane as the test line and control line, respectively, to construct the LFA format. The results were determined by the appearance of the test line and control line (Fig 9). LFA’s sensitivity was assessed using known concentration dilutions of four venoms (0.01–1000 ng/ml) applied in PBS. The assay was completed within 10–15 min, and the results were evaluated by the appearance of red lines at test and control zones. The red lines were not presented at respective test zones when FS, NA, and OH dilution samples of each gradient were checked by the LFA strips, confirming a distinct specificity of LFA. To determine the sensitivity of the LFA strip, we performed five parallel assays using known concentrations (0.01 to 1000 ng/ml) and dilutions of BM venom samples applied to strips, which generated red line at the respective test zone, using PPS as the negative control. The results indicated the sensitivity limit of LFA detecting venom up to 1 ng/ml (Fig 10). All samples (including 90 tissue homogenates, 30 blood samples, and 13 urine samples) taken from envenomed rats displayed positive results. The qualitative results obtained by LFA were compared with sandwich ELISA and are summarized in Table 1.

**Table 1:** Evaluation of LFA and ELISA test for comparing the results from experimentally envenomed samples.

LFA	Time interval in h (ng/ml)				
	1	2	4	6	8
Brain (+)	22.53	26.62	14.51	6.44	2.50
Liver (+)	65.14	96.04	52.56	28.41	8.43
Site of injection (+)	499.12	399.21	275.26	142.4	56.2
Blood (+)	345.14	252.10	176.14	88.41	35.4
Urine (+)	15.26	35.45	18.46	NA	10.45

NA: not applicable

## Discussion

The diversity of snake species presents a threat to public health, with an increasing risk of snakebites and difficulty in clearly distinguishing the responsible snake. Misidentification is common even when a dead snake or its picture is sent to an emergency center [13]. The identification of snake species is beneficial for timely antivenom administration. Therefore, a rapid diagnosis of venomous snakebites is an important means to realize effective antivenom administration.

ELISA is a method based on enzyme-labeled antibodies that can detect antigens immobilized on the surface of a polystyrene plate [48]. Sandwich ELISA shows the highest sensitivity for detecting the specific antigens in complex samples [49], and enables the detection and quantification of venom proteins in various body fluids such as blood, urine, blisters, and wound extracts [19]. Many venom components from the *Elapidae* family share similar amino acid sequences. For instance, venoms from BM and NA contain one common protein family and six less abundant species-specific protein families. Phospholipase A_2_ (PLA_2_) can also be found in NA and FS, accounting for 12.2% and 71% of components in the total protein, respectively [50,51]. Strong cross-reaction was observed between the antivenin and venom from snakes of the genera *Bungarus*, which was more obvious than that between NA and OH (Fig 5A). Therefore, strong similarity in venom component and relative abundance of protein families were found in phylogenetically proximal species. In this study, we assessed the levels of cross-reaction using different immunological methods and found that strong venom × antiserum cross-reactivities between BM antivenin and other three heterogeneous venoms and corresponding cross-reactive antigen molecules were record in the western blot assay (Fig 5). The small toxins had a similar MW distribution in these four venoms transferred to the bottom of SDS-PAGE gel and formed very thick bands (Fig 3), which might have contributed to the results of the cross-reaction. Cross-reactivity was expectable because snake venom proteins have very similar components and biological activities [52]. Heterologous venoms showed extensive cross-reactions to a monovalent or polyvalent antibody, which hampered the specificity of venom detection [53,54]. With the progress in proteomics, as well as in-depth investigation on snake venom-specific proteins and their functions, the use of monoclonal antibody targeting single species-specific venom protein was considered to significantly improve the specificity of detection [55]. A monoclonal antibody possesses only one specific antigen-binding site, commonly binding with high affinity and specificity to only one-antigen epitope [56]. At present, several currently available commercial LFA often produce monoclonal antibody against a specific epitope from a single antigen and then immobilize on the same platform as capture and coating antibodies. However, certain limitations are observed in snake venom detection when a single toxin is used as the antigen; for example, insufficient antigenicity leads to low sensitivity of the produced antibody, and the single toxin may undergo degradation by the biological system. Also, components of snake venom may be influenced by seasonal variation, geographical distribution, or individual size [16,40]. In addition, the antigen–antibody interaction and identification are not simply based on the MW of the protein but with the common antigenic epitope as the recognition target [13,23]. Two antibodies derived from different species can recognize two different antigenic determinants on the antigen molecule, and sandwich ELISA exhibited very high sensitivity and specificity [57]. Sensitivity is a key point for determination of snake venom by immunoassay. Immunoaffinity purification can remove cross-reacting antibody molecules that share a common epitope rather than the whole antibody while retaining non-cross-reacting antibody molecules, which can not only help acquire specificity but also improve the detection sensitivity. Hence, the critical step was preparing SSAb against a specific venom. First, the capture antibody isolated from purified hyperimmune plasma with an affinity purification column (Protein A), and SSAb obtained by affinity chromatography. Thus, high affinity and specific antibodies were prepared and used for sandwich ELISA, and the sensitivity could be further improved by the enzyme amplification effect. The affinity and specificity of SSAb were tested by ELISA and western blot assay (Fig 6), and the results showed that it could detect an extremely low concentration of snake venom with high specificity.

Neurotoxins are the most clinically important toxins in the *Elapidae* family venom, including three-finger toxin (3FTx) and PLA_2_ [50]. The protein profiles in the present study showed strong similarity, and the main protein bands were about 10 kDa, which might be ascribed to a-bungarotoxin being more abundant in these venoms. In addition, the presence of medium proteins bands around 60 kDa is rather clear in venoms of BM and OH (Fig 3). The PLA_2_ and 3TFx toxins were most abundant in the *Elapidae* family, which contains common protein family, may be the cause of cross-neutralization between the monovalent antibody and heterologous snake venoms [50].

Two prior studies found that patients received overdose antivenom for the treatment of *Bothrops* snakebites, which also increased the cost of antivenom treatment as well as the odds of allergic reaction [20,58]. Other studies revealed that the clearance of antivenom was significantly faster than several venom components, thereby causing a rebound of envenomation signs and symptoms [59,60]. Sandwich ELISA can quantitatively detect venom in blood or biological fluid samples, which can allow us to objectively determine the efficacy of antivenom [19]. As is well known, antivenom can effectively bind to circulating snake venom antigens [19]. Consequently, sensitive methods could preferably detect venom level and then appropriately administrate antivenom dosage, saving valuable antivenom and reduce adverse reaction. Taking this into account, snakebite management requires a rapid, simple, and sensitive detection. Even though the limits of quantification of LFA are higher than those of ELISA, the former is easier to operate, more convenient, and requires a shorter runtime than the latter. In this study, serum sample from hyperimmune rabbit, which developed high-titer antiserum (higher than 100,000) containing high affinity antibodies, was applied to antibody purification. The high-affinity APAb and SSAb immobilized on the LFA strip to construct a convenient diagnostic tool. The developed LFA for detecting BM venom was highly sensitive with a minimum detection limit of 1 ng/ml. This finding is comparable with that reported by Pawade et al. [40].

Bao et al. [6] and Mao et al. [38] detected the amount of snake venom in Sprague–Dawley (SD) rats and found that the mean yield of dried BM venom was 4.6 mg after a snakebite. The mean amount of BM venom after a snakebite was 0.414 μg venom/g body weight for SD rats, according to the equivalent dose of conversion between humans and animals based on body surface area. Understanding the biodistribution and kinetics of snake venom can facilitate the understanding of the envenoming process and improve the diagnosis and treatment. In this study, high levels of venom concentrations were demonstrated in blood and the muscle at the site of injection (Fig 8). Also, the detection of urine in early stage was found to be conducive to the envenoming diagnosis (Table 1). This finding is consistent with a previous report that the exudates of the bite site were most suitable for venom assessment [18]. The highest concentration of venom can be detected in tissues in the bitten area of human patients, while the lowest concentration is observed in the brain [26]. Additionally, the venom in blood reached a peak concentration after 1 h, which also indicated that BM venom was rapidly absorbed and entered blood circulation from the bite site, perhaps because neurotoxin is dominated by proteins and enzymes with a low-median MW. These data underscored that early antivenom administration is urgent as antivenom can only neutralize unbound venom antigens [23]. Low concentrations of venom in brain indicated that the blood–brain barrier may play a protective role against venom from entering the brain and displays a low-affinity for the brain tissue. Notably, the detection of venom in vivo largely depends on the time interval and rate of absorption, and re-distribution and degradation of venom occurs over the period [16]. Venom concentrations depend on various factors involving the sensitivity of the assay, the venom dose injected, the rate of venom absorption, individual patient factors, and the time post-bite. In this study, we developed ELISA and LFA to determine BM venom in experimental animals. The results were visualized within 10–15 min, and 100% agreement was observed between LFA and ELISA (Fig 10 and Table 1). Our study has a few limitations, such as the developed strip had not performed with actual snake envenomated patient samples or BM venom from other countries.

In conclusion, distinctive protein antigen venoms may belong to the same family. High sensitivity and ultra-low detection limits were obtained by sandwich ELISA and LFA. The current LFA was easy to perform, less time-consuming, and suitable for snakebite detection and management. The method described in our study with the rapidity and specificity is feasible enough to become a diagnostic tool for discriminating snakebites after further studies and improvement of detection sensitivity.
